# Selective frontal neurodegeneration of the inferior fronto-occipital fasciculus in progressive supranuclear palsy (PSP) demonstrated by diffusion tensor tractography

**DOI:** 10.1186/1471-2377-11-13

**Published:** 2011-01-26

**Authors:** Pia Kvickström, Bengt Eriksson, Danielle van Westen, Jimmy Lätt, Christina Elfgren, Christer Nilsson

**Affiliations:** 1 Geriatric Psychiatry, Department of Clinical Sciences, Lund University, Klinikgatan 22, 22185 Lund, Sweden; 2 Center for Medical Imaging and Physiology, Skåne University Hospital, Lund, Sweden

## Abstract

**Background:**

The clinical presentation in progressive supranuclear palsy (PSP), an atypical parkinsonian disorder, includes varying degrees of frontal dysexecutive symptoms. Using diffusion tensor imaging (DTI) and tractography (DTT), we investigated whether diffusion changes and atrophy of the inferior fronto-occipital fasciculus (IFO) occurs in PSP and if these changes correlate with disease stage and clinical phenotype. The corticospinal tract (CST), which is often involved in PSP, was investigated for comparison.

**Methods:**

DTI of the whole brain was performed with a 3 T MR scanner using a single shot-EPI sequence with diffusion encoding in 48 directions. Scans were obtained in patients with PSP (n = 13) and healthy age-matched controls (n = 12). DTT of the IFO and CST was performed with the PRIDE fibre tracking tool (Philips Medical System). Fractional anisotropy (FA) and apparent diffusion coefficient (ADC) were calculated and correlated with disease stage and clinical phenotype.

**Results:**

In patients with PSP, significantly decreased FA and increased ADC was found in the frontal part of IFO compared with the medial and occipital parts of IFO, as well as compared to controls. Four of the thirteen patients with PSP showed a marked decrease in the number of tracked voxels in the frontal part of IFO. These findings were most pronounced in patients with severe frontal cognitive symptoms, such as dysexecutive problems, apathy and personality change. There was a strong correlation (r^2 ^= -0.84; p < 0,001) between disease stage and FA and ADC values in the CST.

**Conclusions:**

DTT for identification of neuronal tracts with subsequent measurement of FA and ADC is a useful diagnostic tool for demonstrating patterns of neuronal tract involvement in neurodegenerative disease. In selected tracts, FA and ADC values might act as surrogate markers for disease stage.

## Background

Progressive supranuclear palsy (PSP) is a neurodegenerative disorder, with progressive motor, behavioural and cognitive symptoms, ultimately leading to severe handicap and death [1]. It is one of the most common parkinsonian disorders, after idiopathic Parkinson disease (IPD) and dementia with lewy bodies (DLB) [2]. The clinical syndrome initially described by Steele, Richardson and Olszewski [3] is characterized by vertical gaze palsy, early postural instability, axial rigidity, combined with apathy and dysexecutive symptoms, and is referred to as Richardson's syndrome [1]. Recent clinical and neuropathological studies have revealed other clinical presentations of PSP, dominated by bradykinesia and postural instability and lacking many of the frontal behavioural disturbances, so-called PSP-parkinsonism [1].

Although often classified clinically as an atypical parkinsonian disorder, PSP is a tauopathy closely related to corticobasal degeneration (CBD) and therefore also grouped with other tau-positive frontotemporal dementia disorders. The diverse presentations make clinical diagnosis difficult, especially in early stages of the disease, and there is a need for additional tools to support the clinical diagnosis. Magnetic resonance imaging (MRI) has so far proved to be the most useful diagnostic investigation in clinical practice. Routine MRI can demonstrate atrophy of the midbrain, putamen, superior cerebellar peduncle (SCP) and frontal cortex, as well as signal changes in the putamen and SCP [4,5]. The sensitivity and specificity of these signs vary, but none of them are completely specific for PSP [4-6].

MRI with diffusion tensor imaging (DTI) has enabled measurement of the average and directional diffusivity of water molecules in living tissue and has been used for investigations of various brain disorders [7-9]. With appropriate software the diffusion data can be further processed to create a three-dimensional map that represents the white matter pathways. This technique is called diffusion tensor tractography (DTT) and has lately become a valuable tool for studying both healthy and diseased white matter [10]. We have previously demonstrated the potential use of DTT for differential diagnosis of atypical parkinsonian disorders, including PSP [11].

The inferior fronto-occipital fasciculus (IFO) is a large white matter tract connecting the frontal, temporal and occipital lobes. The IFO also constitutes one of the major efferent and afferent neuronal projections to the frontal lobes. It runs from the lateral aspect of the frontal lobe, passing through the extreme and external capsules, as well as the temporal lobe stem, before radiating into the posterior temporal and occipital cortex [12,13]. The functions of the IFO are still poorly understood. Apart from being a major association pathway between the frontal and occipital lobes, there is anatomical evidence that this pathway constitutes a connection between the prefrontal cortex and auditory and visual association cortex in the temporal lobe [13]. This might form a part of the anatomical substrate for the suggested association of the IFO with temporal lobe syndromes, semantic processing, global aphasia, and attentional set-shifting ability, although several other association tracts that connect frontal cortex to more posterior regions are affected in these conditions and cognitive processes as well [13-16].

PSP-Richardson's syndrome serves as a model for neurodegenerative diseases with prominent fronto-subcortical involvement. As IFO connects the frontal lobes with more posterior brain regions, it is therefore of great clinical interest to investigate this pathway in PSP. Although validated diagnostic neuropathological criteria exist for PSP and the distribution of pathological lesions are well described [1,17], the neuropathology of the IFO has not been studied in PSP. Furthermore, the pattern of neuronal tract degeneration underlying the different PSP syndromes has not been characterized. The aim of the present study was therefore to investigate whether atrophy of the IFO occurs in PSP and if there is a correlation between the clinical presentation in PSP and IFO atrophy and diffusion changes. For reference, the corticospinal tract (CST), which is often involved in PSP [17], was chosen for comparison.

## Methods

### Subjects

Thirteen patients with progressive supranuclear palsy (PSP; 8 male, 5 female; age (mean/SD) 70/6) with maximum disease duration of four years were recruited for the study. The diagnosis of probable PSP was made using established clinical criteria [18]. In addition, the following specific symptoms were recorded after clinical examination, review of medical records and interview with caregiver: presence of vertical gaze palsy, severe postural imbalance with falls and clinical fronto-subcortical symptoms (the latter grouped in three categories: dysexecutive symptoms, apathy/lack of initiative and personality change). Disease severity in the PSP patients was categorized using the Schwab & England (S&E) scale for Parkinson's disease [19]. Seven of the subjects with PSP were moderately to severely affected with S&E scale values of 20-40%, while the remaining six subjects had S&E scale values of 60-80%. Twelve healthy age-matched controls (7 male, 5 female; age (mean/SD) 69/8), according to interview and clinical examination, were recruited for comparison. Conventional magnetic resonance imaging (MRI) was used to exclude the presence of vascular or other focal lesions in PSP patients and controls. All patients and controls gave written consent to participate in the study, which was approved by the Regional Ethics Committee for Research.

### Imaging protocol

A 3.0 T Philips Intera MR scanner, equipped with an eight-channel head coil was used. DTI was performed in transversal slice orientation, using a single-shot EPI sequence with diffusion encoding in 48 directions (b values 0 and 800 s/mm^2^). The acquired voxel size was 2 × 2 × 2 mm^3 ^in 50 slices, with a SENSE factor of 2.5. The DTI protocol was followed by a high-resolution T2-weighted sequence for identification of anatomical structures as well as signal changes.

### Neuroradiological evaluation

All T2-weighted images in both patients and controls were evaluated by the same neuroradiologist (DvW). The presence or absence of frontal periventricular or subcortical white matter changes, respectively, as well as the occurrence of atrophy of the frontal cortex and mesencephalon, was recorded. Isolated subcortical white matter lesions <3 mm were regarded as non-significant.

### Tractography

The diffusion-weighted data were transferred to a workstation and a correction algorithm for eddy current correction was applied prior to the tractography [20]. Tractography was then performed using the PRIDE fiber-tracking tool supplied by Philips Medical Systems, as described previously [11].

**Figure 1:**
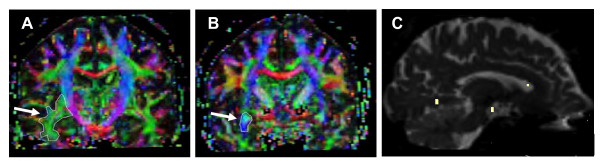
**Regions of interest for tractography of the IFO**. Tractography of the IFO was performed using a two-ROI approach, based on the connection between two regions of interest (ROI; arrows). The two ROIs were placed along the course of the IFO in the coronal plane of the DTI images at the level of the anterior commissure (Figure 1A) and pontine crossing fibers (Figure 1B), respectively. For measurements of regional differences in diffusion parameters, small ROIs were placed in the anterior, middle and posterior segments of the IFO (Figure 1C). The location and size of each ROI is shown in the sagittal plane. For comparison with the location of the IFO, see Figure 3.

Tractography of the IFO and CST bilaterally was performed based on the connection between two regions of interest (ROI) in order to minimize the risk of including other tracts. The atlases by Wakana et al [21] and Catani et al [22] were used to localize the tracts. For tractography of the IFO, two ROIs were placed along the course of the IFO in the coronal plane of the DTI images at the level of the anterior commissure (Figure 1A) and pontine crossing fibers (Figure 1B), respectively [12]. For tractography of the CST, ROIs were placed in the internal capsule and pons, using transversal sections (not shown) [21,22].

To analyze whether diffusion parameters change along the course of the IFO, three small ROIs were fitted to the pathway in cross section, to measure the fractional anisotropy (FA) and the apparent diffusion coefficient (ADC) in frontal (at the level of the genu corpus callosum), middle (by the anterior commissure) and posterior (in the region of the posterior part of corpus callosum) parts of the tract (Figure 1C). Care was taken not to include voxels outside the tract, to avoid partial volume effects. Quantification of FA and ADC in either individual ROIs or whole tract was performed using the PRIDE fiber-tracking tool.

In this study, atrophy of the IFO was defined as a reduction of tracked voxels determined by visual inspection of DTT images. Only patients with a marked reduction of tracked voxels in the frontal segment of the IFO were judged to have significant atrophy.

### Statistical analyses

All the statistical analyses were calculated using SPSS 14.0. The Mann-Whitney U-test was used for comparison of FA and ADC values, respectively, between patients and controls, as well as for different ROIs in the IFO. The mean of the values for the left and right tract ROIs was used. For correlations between disease stage and FA/ADC we used Spearman's rank correlation coefficient. The correlation between clinical fronto-subcortical symptoms (minimum 2/3 of dysexecutive symptoms, apathy/lack of initiative and personality change) and frontal atrophy of the IFO (defined by visual inspection) was evaluated statistically by a 2 × 2 contingency table using Fisher's exact test.

## Results

**Figure 2:**
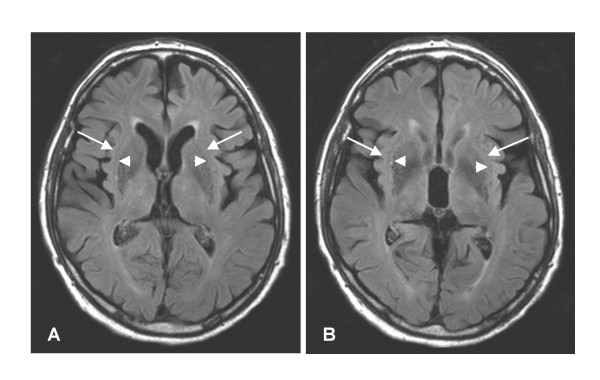
**Axial T2 FLAIR images along the course of the IFO**. MRI T2 FLAIR images, in a patient with PSP and IFO atrophy. The figures show adjacent axial sections (A, B), which include the external capsule (short arrows) and extreme capsule (long arrows). The IFO passes through both these structures. In each section a single minimal white matter signal intensity can be seen in the right external capsule. Note the absence of signal changes in white matter in general, in spite of the presence of mild frontal cortical atrophy.

Visual inspection of the T2-weighted images revealed periventricular white matter changes in 9/13 subjects with PSP, and 4/12 controls. Subcortical white matter changes were only found in one subject with PSP and one control. None of these changes were located along the course of the IFO (Figure 2). Atrophy of the mesencephalon ("humming bird sign") was seen in 9/13 subjects with PSP, while only 4/13 showed frontal cortical atrophy. No regional atrophy was seen in controls.

**Figure 3:**
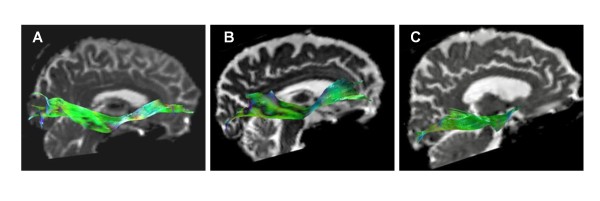
**IFO morphology in patients and controls**. Tractography of the right IFO in a control individual (A), a patient with PSP-parkinsonism (B) and a patient with PSP and dysexecutive problems, apathy and personality change (Richardson's syndrome; C). No difference in IFO morphology can be seen between the control (A) and the patient with PSP-parkinsonism (B), while the patient with PSP-Richardson's syndrome shows severe atrophy of the frontal part of the IFO (C).

**Figure 4:**
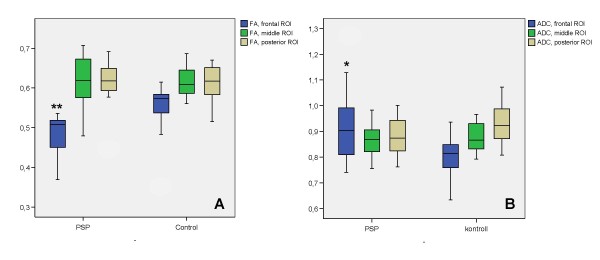
**FA and ADC in the IFO**. Box-plot of FA (A) and ADC (B; mm^2^/s) values in the frontal, middle and posterior ROIs of the IFO (see text for description of location). In the frontal ROI the FA value was significantly decreased (**; p < 0.01), while the ADC value was significantly increased (*; p < 0.05), compared to controls. No difference in either FA or ADC was seen between controls and patients with PSP for the middle or posterior ROIs.

The anatomy of the IFO generated by tractography in the present study (Figure 3), closely resembled previously published data [12,13,16]. On visual inspection 4/13 of the patients with PSP showed a very marked decrease in the number of tracked voxels in the frontal region of the IFO, as illustrated in Figure 3. Diffusion parameters could not be measured in the frontal ROI in two of the 13 subjects with PSP, due to the near absence of tracked voxels in this region. For the remaining 11 patients, a significant difference was seen between PSP and controls for both FA and ADC in the frontal ROI, with lower FA and higher ADC in patients with PSP, while no differences were seen in the middle and posterior ROIs (Figure 4). Comparisons between different ROIs were not performed for the CST. No significant differences in mean FA or ADC, as measured in the whole tract, were seen in the IFO or CST between patients with PSP and controls (Table 1). However, the standard deviation in both FA and ADC was much greater in the IFO than in the CST, in both controls and patients (Table 1).

**Table 1:** FA and ADC values in the IFO and CST.

	PSP		Control	
N	13		12	

	Mean	SD	Mean	SD

FA_IFO_	0.48	0.27	0.49	0.25

ADC_IFO _[mm^2^/s]	0.95	0.53	0.93	0.46

FA_CST_	0.59	0.03	0.59	0.03

ADC_CST _[mm^2^/s]	0.84	0.05	0.81	0.04

Mean and standard deviation (SD) for FA and ADC calculated in whole nerve tracts produced by tractography. There were no statistically significant differences in mean FA and ADC between patients with PSP and controls.

The disease stage, as measured by the Schwab and England scale, was compared with FA and ADC values in the IFO. A significant correlation of 0.58 (p < 0.05) was seen between disease stage and mean FA in the frontal ROI of IFO, while no correlation was seen with the whole IFO or the two posterior ROIs. In addition, a correlation of -0.52 was seen between disease stage and ADC values, but this correlation was not statistically significant (p = 0.07). For the whole CST, there was a highly significant negative correlation between disease stage and ADC (-0.84; p < 0,001), while a statistically non-significant correlation of 0.48 (p = 0.095) was seen for FA values.

In a further analysis, clinical fronto-subcortical symptomatology (at least two out of three of dysexecutive symptoms, apathy and personality change) was compared with the presence of frontal atrophy in the IFO. In patients with a frontal clinical profile, four out of five had significant atrophy of the frontal IFO, as determined by visual inspection, and all of these four patients were in an advanced disease stage (Schwab and England 20-30%). None of the patients that had less than two of the frontal clinical signs listed above showed any atrophy of the IFO. A significant correlation (p < 0.01) was seen between a fronto-subcortical clinical profile and the presence of frontal atrophy of the IFO (Table 2). On routine MRI, all patients with fronto-subcortical symptoms also showed atrophy of the mesencephalon, while only 2/5 showed frontal lobe cortical atrophy.

**Table 2:** Correlation between clinical symptoms and frontal atrophy of the IFO.

	Frontal atrophy		
**Frontal profile**	**Yes**	**No**	**Total**

Yes	4	1	5

No	0	8	8

Total	4	9	13

Correlation between clinical fronto-subcortical symptoms ("frontal profile"; i.e. minimum 2/3 of dysexecutive symptoms, apathy/lack of initiative and personality change) and frontal atrophy of the IFO (defined by visual inspection), using a 2 × 2 contingency table (p < 0.01).

## Discussion

In the present study, we demonstrate that atrophy and diffusion changes occur in two major nerve tracts (IFO and CST) in patients with PSP using aggregated data from DTT performed in individual patients. As the IFO appears to be affected only in some of the patients with PSP and frontal cognitive and behavioral symptoms, this could explain the failure of previous studies to demonstrate changes in diffusion parameters along the IFO [25,27]. This finding further emphasizes the clinical heterogeneity and variable distribution of pathological changes in patients with PSP [1].

Previous imaging studies in PSP and other atypical parkinsonian disorders have mainly focused on the patterns of regional atrophy and their relation to clinical diagnosis [4,5]. Using voxel-based DTI on a group level, significant changes in diffusion parameters have previously been reported in the superior longitudinal fasciculus, anterior corpus callosum, arcuate fasciculus, fornix, posterior thalamic radiation, internal capsule, superior cerebellar peduncle and cerebellar white matter in patients with PSP [25,27]. The IFO is a major cortical association pathway connecting the frontal and occipital lobes, with some branches also terminating in the parietal and temporal lobes [12,16]. The IFO cannot be delineated by conventional MRI, but can easily be visualized using tractography. By using two or more ROIs, it is possible to delineate the IFO and separate it from tracts that are adjacent to the IFO during parts of its course [12]. If the visualized tracts follow the same route as anatomically defined neuronal pathways, it is reasonable to assume that they are a good approximation of this pathway. The anatomy of the IFO generated by tractography in the present study closely resembles previously published tractography and post-mortem dissection data [12,13,16].

It is important to point out that the images of fiber tracts produced by the tractography software probably are an approximation of the underlying neuronal pathways. Each "fiber" is only a representation of voxels that have similar macroscopic diffusion properties. For instance, the DTT model used in this and most other published studies does not differentiate between anterograde and retrograde connections and cannot separate fiber tracts that cross, meet, merge or diverge [10]. Some of these problems can potentially be overcome by using higher orders of tensors or spherical deconvolution [26]. However, these methods are not applicable to the material in the present study.

FA and ADC values obtained in the whole IFO produced by DTT are the mean of a large number of voxels and the risk for partial volume effects are minimized. On the other hand, variations in FA and ADC along the course of the IFO will not be evident. By measuring FA and ADC in small cross-sectional ROIs along the IFO, the mean values will depend on only a few voxels with increased risk for statistical errors. However, by placing these small ROIs within the limits of the tract in the DTI coronal image, the risk for partial volume effects was minimized.

FA and ADC values differ between different tracts [11], probably reflecting differences in axon density as well as the degree of homogeneity of axon type and direction. In this context, it is interesting to note the substantial variation in mean FA and ADC values in the IFO, as shown by the high standard deviation in both patients and controls in Table 1. This might indicate that the IFO has a heterogeneous composition, although other technical reasons could also be involved. In contrast, very little variation was seen in either FA or ADC in the CST, which consists almost entirely of large myelinated primary motor neuron fibers.

It is well known that the CST can be involved in PSP [17] and one third of patients show pyramidal signs [28]. We found no difference in FA or ADC values in the CST between patients with PSP and controls. However, there was a strong correlation between ADC values and disease severity in the CST in our study, and this indicates that involvement of the motor cortex and the CST increases gradually with disease progression in PSP.

In the present study, four out of the five patients with classical PSP-Richardson's syndrome had severe atrophy of the frontal section of the IFO in DTT images, which to our knowledge has not been demonstrated previously in vivo. Neuropathological lesions in PSP can be widespread in frontal, parietal and subcortical areas of the brain, while the occipital lobes usually are unaffected [1,17]. White matter degeneration in PSP is usually moderate and is mainly due to neuronal or axonal degeneration in the more severely affected areas [17]. This is in accordance with the present study, where frontal subcortical white matter changes in routine MRI were found in less than 10% of both subjects with PSP and controls. Therefore, it seems reasonable to assume that frontal atrophy of the IFO is secondary to the neuronal degeneration in the frontal cortex, which occurs in patients with PSP-Richardson's syndrome [1], leaving more posteriorly located parts of the IFO intact. On the other hand, frontal cortical atrophy was only seen with routine MRI in two of the five individuals that showed clinical fronto-subcortical symptoms. This indicates that significant neurodegenerative changes occur in the frontal part of the IFO before macroscopically evident frontal cortical atrophy can be seen.

In recent years it has become clear that the spectrum of clinical presentation in PSP can be highly variable [1]. This is confirmed in the present study, where only five out of thirteen patients with maximum of four years disease duration showed prominent fronto-subcortical involvement, even though they fulfilled criteria for probable PSP. Although there is a clinical rating scale for PSP [29], we chose to use the Schwab and England rating scale for Parkinson's disease as this provides a simple global rating of disease severity. Our results show that disease severity measured in this way correlated with diffusion parameters in both the IFO and CST.

Studies of the IFO using DTI and DTT have previously demonstrated decreased FA values in children with autism spectrum disorders, traumatic brain injury and following treatment with chemotherapy [30-32], while we have not found any studies of the IFO in adult neurodegenerative disease. The atrophy of the frontal IFO observed in this study, correlated strongly with the presence of signs of fronto-subcortical dysfunction, such as dysexecutive problems, apathy and personality change. The present results therefore indicate that atrophy of the frontal IFO is closely related to the cognitive and behavioral symptoms seen in this neurodegenerative disorder. Future studies should investigate the integrity of other fronto-subcortical nerve tracts in PSP, in combination with more detailed neuropsychological evaluation.

DTI has been used for measurement of white matter diffusion properties in a number of diseases, e.g. amyotrophic lateral sclerosis, schizophrenia, and Alzheimer disease [7-9]. Tractography, on the other hand, has mainly been used for neuroanatomical dissection of white matter tracts in vivo in healthy volunteers and patients with brain tumors, while its use in neurodegenerative disease has been rather limited [10,11,23-25]. DTT could prove useful to investigate the correlation between clinical symptoms and patterns of neurodegeneration and atrophy in specific white matter tracts. This might also be important for differential diagnosis, where the detection of specific patterns of atrophy could point to a specific diagnosis when the clinical history, symptoms and signs are ambiguous, for example in the presence of early frontal symptoms. More importantly, the neuroanatomy of subcortical white matter networks underlying cognition and behavior in both normal brain function as well as in different brain diseases could be deduced by combining measures of cognitive and behavioral function with diffusion changes and atrophy of selected tracts visualized by tractography [15].

## Conclusions

In conclusion, we have demonstrated that selective frontal diffusion changes and atrophy of the IFO occurs in PSP, in patients showing a fronto-subcortical clinical phenotype. Furthermore, DTT can be a useful tool for identifying selective patterns of neuronal tract atrophy in neurodegenerative disease, which cannot be detected by routine MRI. In selected tracts, FA and ADC values might act as surrogate markers for disease stage.

## Competing interests

The authors declare that they have no competing interests.

## Authors' contributions

PK performed the tractography and image analysis, statistical analysis and drafted the manuscript. BE was involved in study design, recruited patients and controls and performed the clinical evaluations. DvW developed the MRI protocols and evaluated the routine MRI images. JL developed the MRI protocols and DTI image processing. CE participated in the tractography and image analysis, statistical analysis, evaluation of data and preparation of the manuscript. CN conceived of and designed the study, participated in all data evaluation and wrote the final version of the manuscript. All authors read and approved of the final version of the manuscript.

## Pre-publication history

The pre-publication history for this paper can be accessed here:

http://www.biomedcentral.com/1471-2377/11/13/prepub

## References

[B1] Williams DR Lees AJ Progressive supranuclear palsy: clinicopathological concepts and diagnostic challenges Lancet Neurol 2009 8 270 279 10.1016/S1474-4422(09)70042-0 19233037

[B2] Litvan I Update on epidemiological aspects of progressive supranuclear palsy Mov Disord 2003 18 Suppl 6 43 50 10.1002/mds.10562 14502655

[B3] Steele JC Richardson JC Olszewski J Progressive supranuclear palsy. A heterogeneous degeneration involving the brain stem, basal ganglia and cerebellum with vertical supranuclear gaze and pseudobulbar palsy, nuchal dystonia and dementia Arch Neurol 1964 10 333 359 14107684 10.1001/archneur.1964.00460160003001

[B4] Yekhlef F Ballan G Macia F Delmer O Sourgen C Tison F Routine MRI for the differential diagnosis of Parkinson's disease, MSA, PSP, and CBD J Neural Transm 2003 110 151 169 10.1007/s00702-002-0785-5 12589575

[B5] Sitburana O Ondo WG Brain magnetic resonance imaging (MRI) in parkinsonian disorders Park Rel Disord 2009 15 165 174 10.1016/j.parkreldis.2008.04.033 19059803

[B6] Sung Y Park K Lee Y Park H Shin DJ Park J Oh M Ma H Yu K Kang S Kim YJ Lee B Midbrain atrophy in subcortical ischemic vascular dementia J Neurol 2009 256 1997 2002 10.1007/s00415-009-5226-z 19618301

[B7] Sach M Winkler G Glauche V Liepert J Heimbach B Koch MA Büchel C Weiller C Diffusion tensor MRI of early upper motor neuron involvement in amyotrophic lateral sclerosis Brain 2004 127 340 350 10.1093/brain/awh041 14607785

[B8] Stebbins GT Murphy CM Diffusion tensor imaging in Alzheimer's disease and mild cognitive impairment Behav Neurol 2009 21 39 49 19847044 10.3233/BEN-2009-0234 PMC3010401

[B9] Ardekani BA Tabesh A Sevy S Robinson DG Bilder RM Szeszko PR Diffusion tensor imaging reliably differentiates patients with schizophrenia from healthy volunteers Hum Brain Map 2010 in press 10.1002/hbm.20995 PMC2896986 20205252

[B10] Ciccarelli O Catani M Johansen-Berg H Clark C Thompson A Diffusion-based tractography in neurological disorders: concepts, applications, and future developments Lancet Neurol 2008 7 715 727 10.1016/S1474-4422(08)70163-7 18635020

[B11] Nilsson C Markenroth Bloch K Brockstedt S Lätt J Widner H Larsson EM Tracking the neurodegeneration of parkinsonian disorders - a pilot study Neuroradiology 2007 49 111 119 10.1007/s00234-006-0165-1 17200869

[B12] Catani M Howard RJ Pajevic S Jones DK Virtual *in vivo *interactive dissection of white matter fasciculi in the human brain Neuroimage 2002 17 77 94 10.1006/nimg.2002.1136 12482069

[B13] Kier EL Staib LH Davis LM Bronen RA MR imaging of the temporal stem: anatomic dissection tractography of the uncinate fasciculus, inferior occipitofrontal fasciculus, and Meyer's loop of the optic radiation Am J Neuroradiol 2004 25 677 691 15140705 PMC7974480

[B14] Perry ME McDonald CR Hagler DJ Jr Gharapetian L Kuperman JM Koyama AK Dale AM McEvoy LK White matter tracts associated with set-shifting in healthy aging Neuropsychologia 2009 47 2835 2842 10.1016/j.neuropsychologia.2009.06.008 19540862 PMC2749901

[B15] Catani M Mesulam M The arcuate fasciculus and the disconnection theme in language and aphasia: history and current state Cortex 2008 44 953 961 10.1016/j.cortex.2008.04.002 18614162 PMC2740371

[B16] Martino J Brogna C Robles SG Vergani F Duffau H Anatomic dissection of the inferior fronto-occipital fasciculus revisited in the lights of brain stimulation data Cortex 2010 46 691 699 10.1016/j.cortex.2009.07.015 19775684

[B17] Hauw JJ Agid Y Dickson D Progressive supranuclear palsy (PSP) or Steel-Richardson-Olszewski disease Neurodegeneration: The Molecular Pathology of Dementia and Movement Disorders 2003 Basel: ISN Neuropath Press 103 114

[B18] Litvan I Bhatia KP Burn DJ Goetz CG Lang AE McKeith I Quinn N Sethi KD Shukts C Wenning GK Movement Disorder Society Scientific Issues Report: SIC Task Force Appraisal of Clinical Diagnostic Criteria for Parkinsonian Disorders Mov Disord 2003 18 467 486 10.1002/mds.10459 12722160

[B19] Schwab RS England AC Gillingham FJ, Donaldson IML Projection technique for evaluating surgery in Parkinson's disease Third Symposium on Parkinson's Disease 1969 Edinburgh: E and S Livingstone 152 157

[B20] de Crespigny AJ Moseley ME Eddy current-induced image warping in diffusion-weighted EPI Proceedings of the International Society for Magnetic Resonance in Medicine 1998 Berkeley, CA 661

[B21] Wakana S Jiang H Nagae-Poetscher L van Zijl P Mori S Fiber tract-based atlas of human white matter anatomy Radiology 2004 230 77 87 10.1148/radiol.2301021640 14645885

[B22] Catani M Thiebaut de Schotten M A diffusion tensor imaging tractography atlas for virtual in vivo dissection Cortex 2008 44 1105 1132 10.1016/j.cortex.2008.05.004 18619589

[B23] Nakata Y Sato N Abe O Shikakura S Arima K Furuta N Uno M Hirai S Masutani Y Ohtomo K Aoki S Diffusion abnormality in posterior cingulate fiber tracts in Alzheimer's disease: tract-specific analysis Radiat Med 2008 26 466 473 10.1007/s11604-008-0258-3 18975047

[B24] Zhang Y Schuff N Du AT Rosen HJ Kramer JH Gorno-Tempini ML Miller BL Weiner MW White matter damage in frontotemporal dementia and Alzheimer's disease measured by diffusion MRI Brain 2009 132 2579 2592 10.1093/brain/awp071 19439421 PMC2732263

[B25] Knake S Belke M Menzler K Pilatus U Eggert KM Oertel WH Stamelou M Höglinger GU In vivo demonstration of microstructural brain pathology in progressive supranuclear palsy: A DTI study using TBSS Mov Disord 2010 25 1232 1238 10.1002/mds.23054 20222139

[B26] Tournier JD Calamante F Gadian DG Connelly A Direct estimation of the fiber orientation density function from diffusion-weighted MRI data using spherical deconvolution Neuroimage 2004 23 1176 1185 10.1016/j.neuroimage.2004.07.037 15528117

[B27] Padovani A Borroni B Brambati SM Agosti C Broli M Alonso R Scifo P Bellelli G Alberici A Gasparotti R Perani D Diffusion tensor imaging and voxel based morphometry study in early progressive supranuclear palsy J Neurol Neurosurg Psych 2006 77 457 463 10.1136/jnnp.2005.075713 PMC2077489 16306152

[B28] Litvan I Update on progressive supranuclear palsy Curr Neurol Neurosci Rep 2004 4 296 302 10.1007/s11910-004-0055-z 15217544

[B29] Golbe LI Ohman-Strickland PA A clinical rating scale for progressive supranuclear palsy Brain 2007 130 1552 1565 10.1093/brain/awm032 17405767

[B30] Aukema EJ Caan MW Oudhuis N Majoie CB Vos FM Reneman L Last BF Grootenhuis MA Schouten-van Meeteren AY White matter fractional anisotropy correlates with speed of processing and motor speed in young childhood cancer survivors Int J Radiat Oncol Biol Phys 2009 74 837 843 10.1016/j.ijrobp.2008.08.060 19117694

[B31] Yuan W Holland SK Schmithorst VJ Walz NC Cecil KM Jones BV Karunanayaka P Michaud L Wade SL Diffusion tensor MR imaging reveals persistent white matter alteration after traumatic brain injury experienced during early childhood Am J Neuroradiol 2007 28 1919 1925 10.3174/ajnr.A0698 17905895 PMC4295209

[B32] Kumar A Sundaram SK Sivaswamy L Behen ME Makki MI Ager J Janisse J Chugani HT Chugani DC Alteration in frontal lobe tracts and corpus callosum in young children with autism spectrum disorder Cerebral cortex 2010 20 2103 2113 10.1093/cercor/bhp278 20019145

